# *Ex vivo* manipulation of bone marrow cells to rescue uremia-induced dysfunction for autologous therapy

**DOI:** 10.1186/s13287-015-0108-z

**Published:** 2015-06-11

**Authors:** Cristina Grange, Benedetta Bussolati

**Affiliations:** Department of Medical Sciences, Molecular Biotechnology Center, University of Torino, via Nizza 52, 10126 Torino, Italy; Department of Molecular Biotechnology and Health Sciences, Molecular Biotechnology Center, University of Torino, via Nizza 52, 10126 Torino, Italy

## Abstract

Uremic toxins are known to affect the regenerative properties of tissue-resident and circulating stem cells and thus appear to be a limiting factor for autologous stem cell-based approaches for treating chronic kidney disease. The recent article by van Koppen and colleagues in *Stem Cell Research & Therapy* provides evidence that an *ex vivo* short-term pre-treatment with statins reverts the dysfunction of bone marrow stem cells isolated from rats with renal impairment. Indeed, statin pre-treated cells improved renal function in a model of established chronic kidney disease. Our commentary discusses the potential of this approach in the context of autologous cell therapy and the available knowledge on the mechanisms involved in uremia-induced stem cell dysfunction.

Chronic kidney disease (CKD) is a life-threatening condition characterized by glomerulosclerosis and tubular atrophy, leading to progressive and irreversible loss of renal function. Several of the compounds retained during progressive renal failure, so-called uremic toxins, display oxidative and pro-inflammatory action with systemic detrimental effects. In this context, increasing data in the literature point out that uremic toxins also affect the functional properties of tissue-resident and circulating stem cells, including bone marrow-derived hematopoietic and stromal cells [1–4].

For several reasons, the damaging effect of uremic toxics on stem cells therefore appears to be a major limitation for stem cell-based regenerative medicine approaches for treating CKD. First, the autologous source of stem cells appears to be of reduced therapeutic interest for patients with CKD, as these cells partially lack regenerative potential. In addition, the uremic toxic milieu may affect stem cells isolated from healthy subjects once injected in patients with CKD. The recent article by van Koppen and colleagues [1] in *Stem Cell Research & Therapy* reports an interesting *ex vivo* approach aimed at overcoming the regenerative impairment of bone marrow stem cells (BMCs) isolated from CKD rats (CKD BMCs). The authors provide evidence that an *ex vivo* short-term pre-treatment (2 hours) with the cholesterol-lowering drug pravastatin is able to revert the loss of regenerative properties of CKD BMCs [1]. In fact, pre-treated cells administered in a single dose to rats with an established CKD showed a therapeutic efficacy *in vivo* that was similar to that of BMCs isolated from healthy rats. Statin pre-treated CKD BMCs, but not untreated cells, improved renal function in terms of glomerular filtration rate and effective renal plasma flow increase, reduced urea, and limited the generation of glomerular and interstitial sclerosis [1].

BMCs have been proposed as an attractive approach for cell therapy because of their regenerative efficacy, and the number of clinical trials is consistently increasing. Nevertheless, the possibility of obtaining an autologous source of a large number of cells in the absence of cell expansion in culture makes BMCs a safe and clinically feasible strategy. The experimental approach proposed by van Koppen and colleagues in CKD rats further supports the clinical interest in BMCs, which were effective in a therapeutic administration protocol for rats already presenting renal function impairment (6 weeks after the 5/6 nephrectomy) [1]. Moreover, the *ex vivo* manipulation of stem cells with statins followed an easily applicable procedure. Additional knowledge on the therapeutic mechanisms of BMCs and on the molecular alterations induced by uremic toxins is of interest, as it could provide clinically relevant information to assess stem cell potency before injection and to identify strategies to manipulate stem cells *ex vivo*.

At present, the analysis of molecular alterations induced by uremia was reported only for endothelial progenitor cells (EPCs) [2] and mesenchymal stromal cells (MSCs) [3, 5, 6]. The detrimental effect of toxins on the EPC compartment has been observed even in cases of mild CKD [2]. Uremic toxins, used in concentrations found in patients with CKD, exerted *in vitro* an anti-proliferative effect on EPCs, affecting the mitogen-activated protein kinase pathway, but did not induce EPC apoptosis [7]. Moreover, they inhibited MSC metabolic activity and proliferation and, more relevantly, altered the secretory activity of MSCs in terms of decreased level of vascular endothelial growth factor and transforming growth factor-beta 1 [3]. It could be speculated that uremia induces epigenetic changes in the BMC genome, inducing a premature senescence and an altered secretion profile, as described for MSCs [8, 9].

By analogy with MSCs, paracrine mechanisms possibly account for the therapeutic activity of BMCs in CKD pre-clinical models, as BMCs release cytokines and growth factors that stimulate the endogenous repair and induce the angiogenic process [10]. Indeed, no integration of BMCs into renal parenchyma was observed [10]. However, the analysis of the secretome in CKD and healthy BMCs as well as in statin-treated cells did not significantly differ in terms of released factors, as only the inflammatory chemochine CXCL-5 was decreased after treatment [1]. It is conceivable that other secreted factors, including exosomes and extracellular vesicles, could be involved and modulated by statins.

Statins have been described to ameliorate stem cell functions, such as proliferation, migration, adhesion, and angiogenic ability, on cells isolated from a healthy subject. In addition, they were effective in the prevention of senescence [11]. By analogy, in CKD-derived BMCs, statins appeared to increase migratory properties *in vitro* and to restore therapeutic potential *in vivo* [1]. Besides uremia, systemic diseases such as diabetes and inflammatory conditions as well as advanced age may affect BMC functionality [3, 4]. Therefore, the search for pharmacological strategies to bypass the patient-related dysfunction of stem cells and, in particular, of BMCs appears to be of great interest for autologous therapy. It would be relevant to evaluate whether the effect of statins is restricted to uremia-induced alterations in BMCs or could be extended to other stem cell types and detrimental conditions. Another proposed approach is based on hypoxia preconditioning of BMCs, which rescued the dysfunction of BMCs from aged mice and enhanced their adhesion, survival, and angiogenic potency [12].

In conclusion, strategies of *ex vivo* manipulation of stem cells are required to restore the regenerating efficacy lost under systemic inflammatory conditions, including uremia, to allow their use in an autologous manner. Statins appear to be an easily applicable and functional strategy for the autologous use of BMCs in CKD in pre-clinical models. The validation of this approach in different stem cell types as well as in human cells would be of great clinical interest.

## Abbreviations

BMC: bone marrow stem cell
CKD: chronic kidney disease
EPC: endothelial progenitor cell
MSC: mesenchymal stromal cell
